# Structural and Functional Alterations of the Temporal lobe in Schizophrenia: A Literature Review

**DOI:** 10.7759/cureus.11177

**Published:** 2020-10-26

**Authors:** Arveen Kaur, Deepak M Basavanagowda, Bindu Rathod, Nupur Mishra, Sehrish Fuad, Sadia Nosher, Zaid A Alrashid, Devyani Mohan, Stacey E Heindl

**Affiliations:** 1 Psychiatry and Behavioral Sciences, California Institute of Behavioral Neurosciences & Psychology, Fairfield, USA; 2 Medicine, California Institute of Behavioral Neurosciences & Psychology, Fairfield, USA; 3 Neurology, California Institute of Behavioral Neurosciences & Psychology, Fairfield, USA; 4 Surgery, California Institute of Behavioral Neurosciences & Psychology, Fairfield, USA; 5 Medicine, Avalon University School of Medicine, Willemstad, CUW

**Keywords:** schizophrenia, temporal lobe, mri imaging, schizophrenia and negative symptoms, temporal lobe epilepsy, structural abnormalities, functional studies, temporal lobe and neuro-psychiatric manifestations, temporal lobe and auditory hallucinations, schizophrenia and temporal lobe

## Abstract

Schizophrenia is a severe chronic mental illness leading to social and occupational dysfunction. Our primary focus in this review article was to analyze further the structural and functional alterations of the temporal lobe in patients with schizophrenia, which might contribute to the associated manifestations we often see in this illness. Our goal was to see if there was any correlation between temporal lobe abnormalities, more specifically, alterations in brain volume and specific symptoms such as auditory and language processing, etc. There is a positive correlation between volume alterations and thoughts disorders in the temporal lobe in the majority of studies. However, superior temporal gyrus volume has also been correlated negatively with the severity of hallucinations and thought disorders in some studies. We utilized Medical Subject Heading (MeSH) search strategy via PubMed database in our articles search yielding 241 papers. After the application of specific inclusion and exclusion criteria, a final number of 30 was reviewed. The involvement of the temporal lobe and its gray and white matter volume alterations in schizophrenia is quite apparent from our research; however, the exact mechanism of the underlying biological process is not thoroughly studied yet. Therefore, further research on larger cohorts combining different imaging modalities including volumetry, diffusion tensor, and functional imaging is required to explain how the progressive brain changes affect the various structural, functional, and metabolic activities of the temporal lobe in schizophrenia.

## Introduction and background

Schizophrenia was first considered a discrete mental condition by a German psychiatrist named Dr. Emil Kraepelin in 1887. Kraepelin used the term “dementia praecox” to describe it and contrasted it with bipolar disorders and other forms of mood disorders [1]. The mean prevalence of schizophrenia is 4.6/1000 for point prevalence and 4.0 for lifetime prevalence. This disorder can develop as early as five years; however, it is infrequent in childhood, and the risk of schizophrenia is equal in both genders [2]. Schizophrenia is a chronic mental disorder that consists of positive, negative, and cognitive symptoms. Positive symptoms include hallucinations, delusions, and disorganized speech and behaviour. Negative symptoms include flat affect, alogia, and avolition, while cognitive symptoms consist of impairment of memory, reasoning, and auditory perception [3,4].

Regarding the cause of schizophrenia, it still remains a matter of debate. However, multiple factors, including genetics, viruses, or early brain damage, can disrupt the functional circuit of neurotransmission in various parts of the brain, i.e., forebrain, hindbrain, and limbic system. This article will discuss how various underlying structural, and functional changes seen on the temporal lobe's magnetic resonance imaging (MRI) study cause the predominant schizophrenia symptoms, i.e., auditory hallucinations and cognitive symptoms [5]. Findings on MRI studies show that functional hyperactivity in the superior temporal cortex increases blood flow during an auditory hallucination episode, suggesting structural abnormalities in schizophrenia [6]. Studies also reported a smaller anterior superior temporal gyrus (STG) volume in patients with schizophrenia than in controls. Thus the various temporal lobe abnormalities cause the characteristic deficits of auditory and language processing in schizophrenia [7]. The excitatory amino acid transporter in the temporal lobe also causes an alteration in glutamate neuronal transmission [8]. Impaired cognitive empathy is a core social cognitive deficit in schizophrenia associated with negative symptoms and social functioning. Medial prefrontal and temporal brain regions show linkage to cognitive impairment as well as negative symptoms of schizophrenia. These findings support the notion of structural abnormalities in the temporal lobe contributing to schizophrenia's pathophysiology [9].

Significantly, the association of schizophrenia with the temporal lobe is still less known. The authors conducted various studies about the association of changes in the temporal lobe contributing to schizophrenia's pathophysiology. However, the facts about which parts of the temporal lobe are most commonly involved in various neuropsychiatric manifestations of schizophrenia are still not studied thorougly. Whether the structural damage exacerbates the symptoms and if it does what is the sequence of harm is uncertain. Also, what kind of proteins involved in synaptic transmission in the neuron of a schizophrenic patient's temporal lobe are also not known yet.

## Review

Temporal lobe and schizophrenia (anatomical structure)

**Figure 1:**
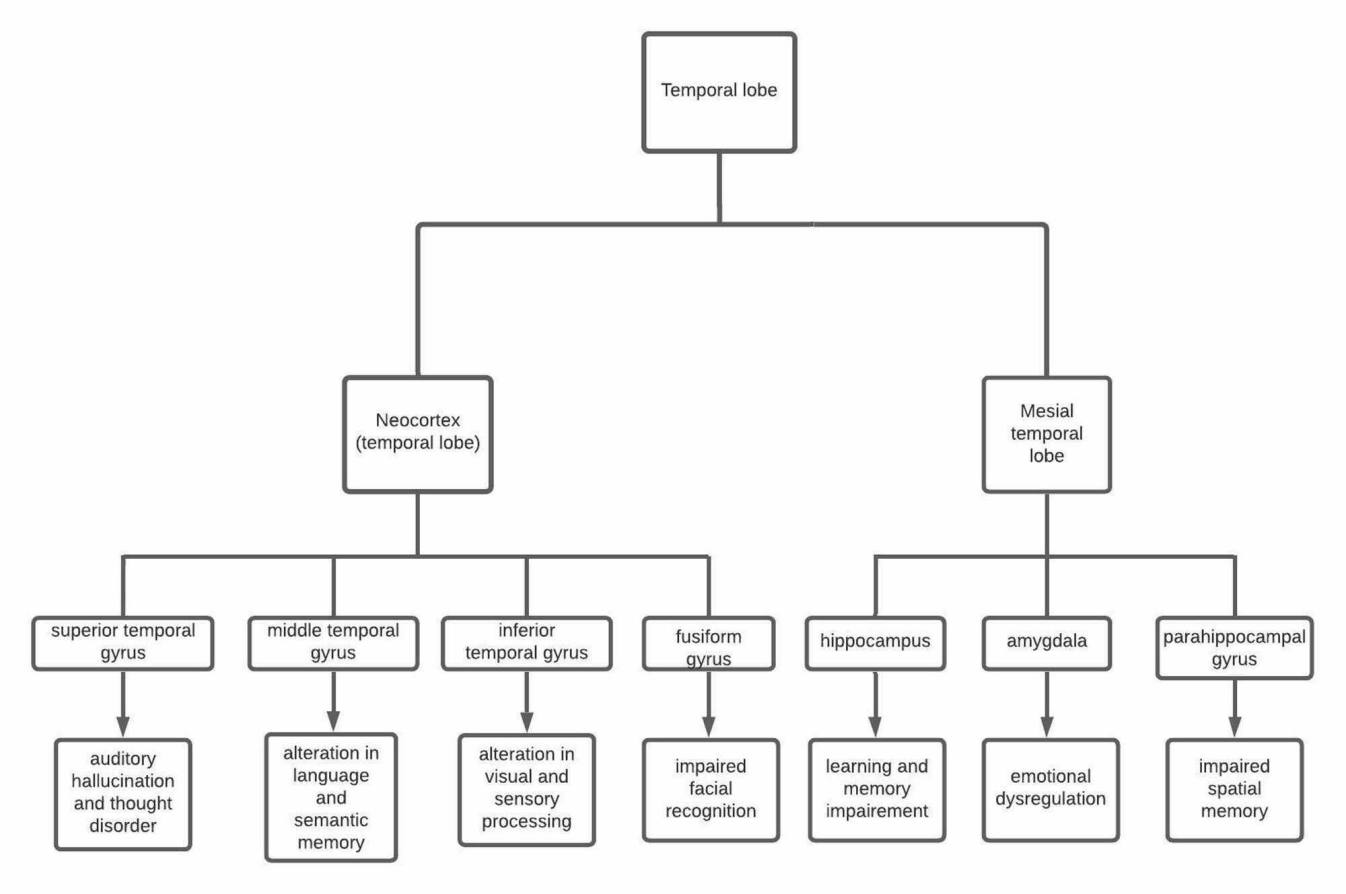
Flowchart showing temporal lobe anatomy and its manifestations

The temporal lobe of the brain is referred to as a neocortex. It forms the cerebral cortex in conjunction with the occipital lobe, frontal lobe, and parietal lobe. The temporal lobe is delineated above by a lateral sulcus (Sylvian fissure) and containing an extension of the lateral ventricle on the lateral surface. Sylvian fissure is a deep cleft but not a fissure in anatomical line. The lobe's lateral surface is indented by superior and inferior sulci, which delineates superior, middle, and inferior temporal gyri. The portion of these curves around into a low surface is called the lateral occipitotemporal gyrus. The temporal lobe's superior surface forms the lateral sulcus floor with the superior temporal gyrus, consisting of Brodmann's area 41 and 42, also known as the primary auditory cortex. The posterior part of the temporal lobe blends into the parietal lobe above and the occipital lobe behind. The midpoint of line drawing from the parieto-occipital sulcus and pre-occipital notch, an indentation in the inferior gyrus, separates the temporal lobe from the parietal lobe. The limbic cortex part is medial inside the temporal lobe's surface, including parahippocampal gyri, hippocampal, uncus, and amygdala [10]. The planum temporale is a triangular area situated on the superior temporal gyrus, referred to as the Wernicke area. Males have a larger left planum temporale showing significant interaction of gender differences and hemisphere [11]. The temporal lobe has six major association fiber tracts. These are uncinate fasciculus, inferior longitudinal fasciculus, inferior frontal occipital fasciculus, middle longitudinal fasciculus, and arcuate fasciculus cingulum. Moreover, the two primary commissural fibers are the corpus callosum and anterior commissure [12]. The demonstration of temporal lobe parts and its various neuropsychiatric manifestations are shown in Figure 1.

Temporal lobe lesions and their neuropsychiatric manifestations

Temporal Lobe Epilepsy

Temporal lobe epilepsy is a disorder of the nervous system characterized by recurrent focal seizures in the temporal lobe. The most common origin of epilepsy is in the hippocampus, called mesial temporal lobe epilepsy. Electroencephalogram (EEG) of mesial temporal limb epilepsy contains interictal features associated with anterior temporal epilepsy discharges. Its characteristics are hippocampal sclerosis, neuronal loss, gliosis, and mossy fiber sprouting [13]. Temporal lobe epilepsy is mainly presenting with acute psychosis symptoms, especially olfactory hallucinations, anxiety, and aggressive behavior [14]. The interictal psychotic syndromes, often resembling schizophrenia, develop in some patients with schizophrenia [15]. The other most frequent sign seen is cognitive impairment. Both early-onset, as well as chronic uncontrolled seizures can add to the initial impairment. The effects of status epileptic can also develop global amnesia, but there is reversible impairment if the episodes are well-controlled [16-18].

Temporal Lobe Infarction

The temporal lobe is involved in social and emotional functions such as the perception of emotional facial expression, sarcasm detection, gaze directions, perception of affective empathy toward others. There is an activation of the superior temporal sulcus and temporal pole areas in sarcasm detection. In temporal lobe infarction, patients exhibit impaired emotional recognition, a deficit in theory of mind, and executive function [19-21]. The study also found that temporal lobe recruitment during an understanding of negative emotions is dependent on the personality of the individuals [22]. The theory of mind is the inferences about other mental states and cognitive capacity that underlies the human ability to engage in complex social interaction. There is a contribution of the frontal lobe and the temporal lobe in the theory of mind. It was reported that there is a deficit in theory of mind after temporal lobe infarction. The traumatic brain injury in the temporal lobe and various psychiatric disorders are accompanied by deficits in theory of mind, which leads to a low social function [23,24].

Kluver Bucy Syndrome

Kluver Bucy syndrome is a type of essential temporal-limbic syndromes. The bilateral anterior temporal lobe causes the syndrome. Kluver Bucy syndrome clinical features include hyperorality, sexual behaviour changes, and psychic blindness [25]. It might be due to disturbances of temporal portions of neurons that interface with multiple cortical and subcortical circuits to modulate emotional behavior and affect. There is a development of complete Kluver Bucy syndrome following left anterior temporal lobectomy. However, the treatment of this syndrome is challenging and unsatisfactory [26].

Auditory Processing in Schizophrenia

The individual with schizophrenia demonstrates impairment in auditory information and higher-order cognitive processing. Functional imaging revealed reduced activity in the limbo and subcortical regions during automatic auditory processing. The findings in schizophrenia are abnormalities in the neural systems associated with processing salient stimuli in the context of oddball auditory tasks. This deficit is also related to abnormalities in orienting, attention, and memory processes [27]. Decreased perfusion in the frontal, temporal, and striate-thalamic region is characteristic of schizophrenia. These dysfunctional circuits may disturb input and output processing, leading to auditory hallucinations [28].

The temporal lobe in schizophrenia

Schizophrenia is characterized by hallucinations, delusions, disorganized speech, behavior, and negative symptoms leading to social and occupational dysfunction. For diagnosing schizophrenia, according to the fifth edition of the Diagnostic and Statistical Manual of Mental Disorders (DSM-5), the patient must have experienced at least two symptoms: hallucinations, delusions, disorganized speech, disorganized or catatonic behavior, and negative symptoms. At least one must be the presence of delusions, hallucinations, and disorganized thought. It must be present for at least six months. Schizophrenia subtype like catatonia and paranoid is not included in DSM-5 by the American Psychiatric Association (APA) as they did not help predict clinical treatment response. There is no actual laboratory test to diagnose this; however, we believe that structural and functional imaging could be future diagnostic modalities for an accurate schizophrenia diagnosis.

Physiological Changes of the Temporal Lobe in Schizophrenia (Structural Imaging)

It is evident from various studies to justify volume reduction (structural changes) in the temporal lobe of schizophrenic patients. In Shenton et al., there was a localized volume reduction of the left temporal lobe and its correlation in thought disorder in schizophrenic patients. In comparison to healthy volunteers, the schizophrenic patients significantly showed reduced gray matter volume in the left anterior hippocampus-amygdala complex and the left superior temporal gyrus. The degree of thought disorder is related to lowering the magnitude of the left posterior superior temporal gyrus [29].

However, in the 20th century, a smaller anterior STG in patients with schizophrenia than in controls was demonstrated. The STG volume has been found to correlate negatively with the severity of hallucinations and thought disorder. These data are consistent with the proposition depicting dysfunction of the primary auditory cortex in the anterior and middle STG and auditory association cortex in the posterior STG. These areas may play a role in producing auditory perceptual abnormalities and poor organization of thought, respectively [30]. A cross-sectional study was conducted used diffusion tensor imaging (DTI) geometric indices. The Voxel-based morphometry (VBM) analysis revealed that patients with first-episode schizophrenia had lower white matter volume in the right temporal-occipital region. Fractional anisotropy (FA) in the implicated region found correlation with the severity of delusions. However, this study suggested that changes in FA may not reflect the underlying pathophysiological processes of cerebral white matter volume reduction, for which further research needed to understand better the nature of white matter changes and its progression in schizophrenia over the period of time [31].

The correlations between structural measures and memory performance in schizophrenia subjects and their siblings was studied. The findings of this study displayed cortical thinning of the middle temporal lobe (MTL) in schizophrenic patients and their siblings as compared to control groups. It was suggested that disease-specific genetic factors might be present in both patients and their relatives, responsible for correlated abnormalities of MTL structure and memory impairment [32]. In comparison to healthy volunteers, there was remarkably lower fractional anisotropy in the temporal (superior temporal and parahippocampal) and occipital (superior and middle the occipital) white matter in schizophrenic patients. In contrast, both patient groups demonstrated significantly higher mean diffusion in frontal and temporal (superior temporal and parahippocampal) gray matter than healthy volunteers but did not differ from each other. Moreover, the temporal and the occipital lobe white matter shortfall can also exacerbate the chances of acquiring schizophrenia [33].

The structural magnetic resonance images for antecedents of schizophrenia (ASz) children and typical developing (TD) children were studied. ASz children showed significantly decreased gray matter (GM) volume in the right middle temporal gyrus (MTG) as well as increased GM volume in the left superior, middle temporal gyri relative to the TD group (typical developing children). These findings might predict the structural brain abnormalities associated with schizophrenia in putatively at-risk, preprodromal children [34]. There was an association of long duration of untreated psychosis with the temporal and occipitotemporal gray matter volume decrease in treatment-naive (treated for the first time) schizophrenia [35]. Another study also demonstrated the reduced gray matter volume in the frontal, temporal, parietal, and occipital lobes of schizophrenic patients. However, earlier onset of psychosis, especially in schizophrenic patients, is associated with the most premature disease-related disruption of anatomical brain growth [36].

The MRI scan demonstrated significantly smaller bilateral STG volumes than healthy subjects. Of the five sub-regions in the STG, patients with schizophrenia showed considerably reduced volumes in the STG and planum temporale (PT) of the STG bilaterally compared with healthy subjects [37]. Another study demonstrated a significant interaction between the group and verbal learning about temporal lobe thickness in relatives of schizophrenic patients, indicating that cortical thickness in the temporal cortex may represent a structural correlate for verbal encoding information in unaffected relatives of individuals with schizophrenia [38]. Cui and his team used 3T T1 weighted brain scan and whole-brain vertex-wise to compare cortical thickness across the group. The conducted study suggested the left middle temporal gyrus involvement in the pathogenesis of auditory verbal hallucinations (AVH), which is the most common and severe symptom of schizophrenia. In schizophrenic patients with AVH, the left middle temporal gyrus was found significantly thinner than in patients without AVHs and healthy controls [39]. Table 1 summarizes structural imaging studies showing the correlation of the temporal lobe with schizophrenia.

**Table 1:** Structural imaging studies showing a correlation of the temporal lobe with the schizophrenia

AUTHOR	YEAR	SAMPLE SIZE	CONCLUSION
Shenton et al. [29]	1992	15-cases 15-controls	Schizophrenia involves localized gray matter volume reduction of the left temporal lobe. The size of the volume reduction in superior temporal gyrus is related to the degree of thought disorder.
Rajarethinam et al. [30]	2000	20-cases 20- controls	The STG (superior temporal gyrus) volume has been found to correlate negatively with the severity of hallucinations and thought disorder.
Chan et al. [31]	2010	39-cases 64-controls	The VBM (voxel-based morphometry) analysis revealed that patients with first-episode schizophrenia had lower white matter volume in the right temporal-occipital region corresponding to the inferior longitudinal fasciculus.
Karnik-Henry et al. [32]	2012	72-cases 97-control	The disease-specific genetic factors may be present in both patients and their relatives, responsible for the correlation of MTL (middle temporal gyrus) structure abnormalities and memory impairment.
Anderson et al. [33]	2013	55-cases 56-controls	The temporal and the occipital lobe white matter shortfall can also exacerbate the chances of acquiring schizophrenia.
Cullen et al. [34]	2013	20-cases 20-control	It indicates that structural brain abnormalities associated with schizophrenia may be detected in putatively at-risk, pre-prodromal children.
Guo et al. [35]	2013	57-cases 30-control	The temporal and occipitotemporal gray matter volume reduction in treatment-naive (treated for the first time) schizophrenia is associated with a long untreated psychosis duration.
Tordesillas-Gutierrez et al. [36]	2015	101-cases 69-controls	The earlier onset of psychosis, especially in schizophrenic patients, is associated with the most premature disease-related disruption of anatomical brain growth.
Ohi et al. [37]	2016	40 –cases 40-controls	Schizophrenic patients had significantly smaller bilateral STG (superior temporal gyrus) volumes than healthy subjects.
Fernandez et al. [38]	2018	62-cases 70-controls	The relatives of schizophrenic patients demonstrated a significant interaction between the group and verbal learning concerning temporal lobe thickness.
Cui et al. [39]	2018	208-cases 261-controls	This study suggested the left middle temporal gyrus involvement in the pathogenesis of auditory verbal hallucinations.

Functional Temporal Lobe Abnormalities in Schizophrenia (fMRI)

Evidence supports various studies suggesting schizophrenia is associated with a reduced left and increased right temporal cortical response to the auditory perception of speech. The patient comparison revealed reduced responsivity of the temporal cortex, specifically to the right middle temporal gyrus, to an external speech during the former state when experiencing severe and mild hallucinations. Thus functional magnetic resonance imaging (fMRI) findings depicting that auditory hallucinatory state is associated with reduced activity in temporal cortical regions [40].

It was suggested that brain pathology expression differs between left-handed and right-handed schizophrenic men and that the pathology is related to the cognitive disturbance. The significant findings revealed bilaterally smaller gray matter volumes in the posterior superior temporal gyrus and the right superior temporal gyrus. This study shows a positive correlation between tissue volume in the right anterior superior temporal gyrus and thought disorder [41]. There was increased medial temporal lobe activation during the passive viewing of emotional and neutral facial expressions in schizophrenia. Relative to control subjects, patients demonstrated significantly greater activation of the left hippocampus while viewing all facial expressions. There was increased right amygdala activation also during the initial presentation of fearful and neutral facial expressions [42].

A study conducted by Goghari et al. investigated whether temporal lobe structural abnormalities were associated with facial emotion recognition deficits in schizophrenia and related to genetic liability for the disorder. In this study, schizophrenic patients exhibited smaller hippocampal and middle temporal volumes. Patients could not improve facial emotion recognition performance with unlimited time to judge but could improve age recognition performance. The study's result suggested a specific deficit in emotion recognition and not attributable to a generalized impairment in face perception. So impaired emotion recognition serves as a target for further interventions [43]. On functional imaging, early-onset schizophrenia (EOS) patients demonstrated increased activation in the anterior cingulate cortex (ACC), medial temporal lobe structures, the insula, and bilateral lateral temporal lobes. The behavioral results showed deficits in EOS patients at all three working memory (WkM) loads. However, it did not found that EOS patients had activation differences in the frontal cortical regions. It supports growing evidence that EOS patients have aberrations in the limbic and temporal lobe regions [44].

There was reduced functional connectivity between planum temporale and temporal, parietal, limbic, and subcortical regions in schizophrenic patients and relatives compared to controls that predisposed towards hallucinations in both patients and relatives. The functional asymmetry of the superior temporal gyrus in patients and relatives, which correlated significantly with the acute severity of hallucinations in the patient group, was observed to be reduced [45]. Another study findings indicate a close relationship between functional and anatomical dysconnectivity in patients with schizophrenia. It might be due to disturbance in the left frontotemporal tracts integrity [46]. A functional connectivity analysis resulted in a left-dominant temporal-frontal network that included speech-related auditory and motor regions. It showed hyper-coupling in the past week, hallucinating schizophrenia patients (relative to non-hallucinating patients) during speech perception. This study opens the possibility that practicing control over inner verbal thought processes may decrease hallucinations' likelihood or severity [47].

The effect of transcranial direct stimulation (tDCS) on the resting-state functional connectivity (rs-FC) of the left temporoparietal junction (TPJ) was investigated. Active tDCS (2 mA, 20 min) reduces resting-state functional connectivity of the left temporal-parietal junction with the left anterior insula. Active tDCS significantly reduced AVH and the negative symptoms relative to sham tDCS (2 sessions/day for 5day). These findings suggest that the reduction of AVH induced by tDCS is associated with a modulation of the rs-FC within an AVH-related brain network, including brain areas involved in inner speech production and monitoring [48]. A recent study also demonstrated that in patients with AVH, there is a decrease in connection from the left inferior frontal gyrus to the left middle temporal gyrus compared to those without AVH. It indicates that the hypoconnectivity or disrupted relationship from frontal to temporal speech areas might be critical for the pathological basis of AVHs. The neuroimaging studies have shown the association of AVHs with altered functional and structural connectivity within the language network [49]. Table 2 summarizes functional imaging studies showing the correlation of the temporal lobe with schizophrenia.

**Table 2:** Functional imaging studies showing correlation of temporal lobe with schizophrenia

AUTHOR	YEAR	SAMPLE SIZE	CONCLUSION
Woodruff et al. [40]	1997	Cases-15 Controls-8	Auditory hallucinations are associated with reduced activity in temporal cortical regions.
Holinger et al. [41]	1999	Cases-8 Controls-10	This study showed a positive correlation between thought disorder and tissue volume in the right anterior superior temporal gyrus.
Holt et al. [42]	2006	cases-15 controls-16	In schizophrenia, elevated hippocampal and amygdala activity during the passive viewing of human faces.
Goghari et al. [43]	2011	Cases-23 Controls- 36	Temporal lobe abnormalities and emotion recognition deficits are prominent features of schizophrenia.
White et al. [44]	2011	Cases-22 Control-24	The behavioral results demonstrated deficits in EOS (early-onset schizophrenia)patients at all working memory.
Ortel-Knöchel et al. [45]	2013	Cases- 46 Controls- 24	Reduced functional connectivity between Planum temporale and temporal, parietal, limbic, and subcortical regions in SZ (schizophrenic) patients.
Leroux et al. [46]	2014	Cases- 20 Controls-20	Results showed left frontotemporal dysconnectivity within the language network in schizophrenic patients, indicating a close relationship between anatomical and functional disconnectivity.
Lavigne et al. [47]	2015	Cases- 45 Controls-27	Hypercoupling in past-week hallucinating schizophrenia patients (relative to non-hallucinating patients) during speech perception.
Mondino et al. [48]	2016	Cases- 23 Controls- 23	Active tDCS (transcranial direct stimulation) significantly reduced AVH (audio verbal hallucinations) and the negative symptoms.
Zang et al. [49]	2017	Cases- 36 Controls- 37	In patients with audio-verbal hallucinations (AVH), there is a decrease in connectivity from the left inferior frontal gyrus to the left middle temporal gyrus.

Limitations

This paper's main limitation was that very few studies performed on temporal lobe parts and its structural and functional abnormalities associated with schizophrenia on which we could base our review. Sufficient extensive data are necessary from the earliest schizophrenia phase to conclude the temporal lobe's alteration in schizophrenia. Moreover, the selected studies' inconsistencies could be partially explained by the MRI data acquisition at various field strengths, i.e., 1.5 Tesla (T) versus 3 T in more recent studies and different morphometric measurements used in various other studies. Therefore, a better understanding will further expand our knowledge and understanding regarding advanced diagnosis modalities.

## Conclusions

In conclusion, there is an association between structural and functional alterations of the temporal lobe with various neuropsychiatric manifestations of schizophrenia. Most of the structural and functional MRI modalities show a positive correlation between thought disorder and volume alteration in the right anterior superior temporal gyrus. However, superior temporal gyrus volume has also been correlated negatively with the severity of hallucinations and thought disorder in some studies. This study also suggested the involvement of the middle temporal gyrus in the underlying pathogenesis of auditory hallucinations. The various temporal lobe abnormalities cause the characteristic deficits of auditory and language processing in schizophrenia. Moreover, the temporal lobe white matter shortfall can also exacerbate the chances of acquiring schizophrenia.

The conclusion emerging from this review is useful in further studies, especially on temporal lobe changes in schizophrenic patients. Even though the exact nature of the underlying metabolic and biological process and potential interaction between different brain regions remain elusive, we must continue to search for better research methodologies to understand the structural basis of schizophrenia. Therefore, further studies on larger cohorts, combining different imaging modalities including volumetry, diffusion tensor, and functional imaging, are needed to elucidate how the progressive brain changes affect the structural, functional, and metabolic activities of the temporal lobe in schizophrenia.

## References

[1] Geraud M ( 2007). Emil Kraepelin: a pioneer of modern psychiatry. on the occasion of the hundred and fiftieth anniversary of his birth. Encephale.

[2] Bhugra D (2005). The global prevalence of schizophrenia. Plos Med.

[3] Milan MJ, Fone K, Steckler T, Horan WP (2014). Negative symptoms of schizophrenia: clinical characteristics, pathophysiological substrates, experimental models, and prospects for improved treatment. Eur Neuropsychopharmacol.

[4] Stepnicki P, Kondej M, Kaczor AA (2018). Current concepts and treatments of schizophrenia. Molecules.

[5] Hugdahl K, Loberg EM, Specht K, Steen VM, Wageningen HV, Jorgensen HA (2008). Auditory hallucinations in schizophrenia: the role of cognitive, brain structural and genetic disturbances in the left temporal lobe. Front Hum Neurosci.

[6] Suzuki M, Yuasa S, Minabe Y, Murata M (1993). Left superior temporal blood flow increases in schizophrenic and schizophreniform patients with auditory hallucination: a longitudinal case study using 123I-IMP SPECT. Eur Arch Psychiatry Clin Neurosci.

[7] Anderson JE, Wible CG, McCarley RW, Jakab M, Kasai K, Shenton ME (2002). An MRI study of temporal lobe abnormalities and negative symptoms in chronic schizophrenia. Schizophr Res.

[8] Shan D, Lucas EK, Drummond JB, Haroutunian V, Meador-Woodruff JH, McCullumsmith RE (2013). Abnormal expression of glutamate transporters in temporal lobe areas in elderly patients with schizophrenia. Schizophr Res.

[9] Abram SV, Wisner KM, Fox JM (2017). Fronto-temporal connectivity predicts cognitive empathy deficits and experiential negative symptoms in schizophrenia. Hum Brain Mapp.

[10] Kiernan JA (2012). Anatomy of the temporal lobe. Epilepsy Res Treat.

[11] Kulynych JJ, Vladar K, Jones DW, Weinberger DR (1994). Gender differences in the normal lateralization of the supratemporal cortex: MRI surface-rendering morphometry of Heschl's gyrus and the planum temporale. Cereb Cortex.

[12] Bajada CJ, Haroon HA, Azadbakht H, Parker GJM, Lambon Ralph MA, Cloutman LL (2017). The tract terminations in the temporal lobe: their location and associated functions. Cortex.

[13] Tatum OW (2012). Mesial temporal lobe epilepsy. J Clin Neurophysiol.

[14] Kanner AM (2004). Recognition of the various expressions of anxiety, psychosis, and aggression in epilepsy. Epilepsia.

[15] Marsha L, Sullivan EV, Morrell M, Lim KO, Pfefferbaum A (2001). Structural brain abnormalities in patients with schizophrenia, epilepsy, and epilepsy with chronic interictal psychosis. Psychiatry Res.

[16] Hermann BP, Seidenberg M, Schoenfeld J, Davies K (1997). Neuropsychological characteristics of the syndrome of mesial temporal lobe epilepsy. Arch Neurol.

[17] Kandratavicius L, Peixoto-Santos JE, Monteiro MR (2015). Mesial temporal lobe epilepsy with psychiatric comorbidities: a place for differential neuroinflammatory interplay. J Neuroinflammation.

[18] Helmstaedter C, Kurthen M, Lux S, Reuber M, Elger CE (2003). Chronic epilepsy and cognition: a longitudinal study in temporal lobe epilepsy. Ann Neurol.

[19] Wong C, Gallate J (2012). The function of the anterior temporal lobe: a review of the empirical evidence. Brain Res.

[20] Kramer UM, Mohammadi B, Donamayor N, Samii A, Munte TF (2010). Emotional and cognitive aspects of empathy and their relation to social cognition--an fMRI-study. Brain Res.

[21] Uchiyama H, Seki A, Kageyama H, Saito DN, Koeda T, Ohno K, Sadato N (2006). Neural substrates of sarcasm: a functional magnetic-resonance imaging study. Brain Res.

[22] Jimura K, Konishi S, Miyashita Y (2009). Temporal pole activity during perception of sad faces, but not happy faces, correlates with neuroticism trait. Neurosci Lett.

[23] Xi C, Zhu Y, Zhu C, Song D, Wang Y, Wang K (2013). Deficit of theory of mind after temporal lobe cerebral infarction. Behav Brain Funct.

[24] Stone VE, Baron-Cohen S, Knight RT (1998). Frontal lobe contributions to the theory of mind. J Cogn Neurosci.

[25] Lanska DJ (2018). The Klüver-Bucy Syndrome. Front Neurol Neurosci.

[26] Ghika-Schmid F, Assal G, De Tribolet N, Regli F (1995). Klüver-Bucy syndrome after left anterior temporal resection. Neuropsychologia.

[27] Kiehl KA, Stevens MC, Celone K, Kurtz M, Krystal JH (2005). Abnormal hemodynamics in schizophrenia during an auditory oddball task. Biol Psychiatry.

[28] Andreasen NC, O'Leary DS, Flaum M, Nopoulos P, Watkins GL, Boles Ponto LL, Hichwa RD (1997). Hypofrontality in schizophrenia: distributed dysfunctional circuits in neuroleptic-naïve. Lancet.

[29] Shenton ME, Kikinis R, Jolesz FA (1992). Abnormalities of the left temporal lobe and thought disorder in schizophrenia. N Engl J Med.

[30] Rajarethinam RP, DeQuardo JR, Nalepa R, Tandon R (2000). Superior temporal gyrus in schizophrenia: a volumetric magnetic resonance imaging study. Schizophr Res.

[31] Chan WY, Yang GL, Chia MY, Lau IY, Sitoh YY, Nowinski WL, Sim K (2010). White matter abnormalities in first-episode schizophrenia: a combined structural MRI and DTI study. Schizophr Res.

[32] Karnik-Henry MS, Wang L, Barch DM, Harms MP, Campanella C, Csernansky JG (2012). Medial temporal lobe structure and cognition in individuals with schizophrenia and their nonpsychotic siblings. Schizophr Res.

[33] Anderson D, Ardekani BA, Burdick KE, Robinson DG, John M, Malhotra AK, Szeszko PR (2013). Overlapping and distinct gray and white matter abnormalities in schizophrenia and bipolar I disorder. Bipolar Disord.

[34] Cullen AE, De Brito SA, Gregory SL, Murray RM, William SC, Hodgkins S, Laurens KR (2013). Temporal lobe volume abnormalities precede the prodrome: a study of children presenting antecedents of schizophrenia. Schizophr Bull.

[35] Guo X, Li J, Wei Q (2013). Duration of untreated psychosis is associated with temporal and occipitotemporal gray matter volume decrease in treatment naïve schizophrenia. Plos One.

[36] Tordesillas-Gutierrez D, Koutsouleris N, Roiz-Santianez R (2015). Grey matter volume differences in non-affective psychosis and the effects of age of onset on grey matter volumes: a voxelwise study. Schizophr Res.

[37] Ohi K, Matsuda Y, Shimada T (2016). Structural alterations of the superior temporal gyrus in schizophrenia. Eur Psychiatry.

[38] Fernandez VG, Asarnow R, Narr KL, Subotnik KL, Kuppinger H, Fogelson D, Nuechterlein KH (2018). Temporal lobe thickness and verbal memory in first-degree relatives of individuals with schizophrenia. Schizophr Res.

[39] Cui Y, Liu B, Song M (2017). Auditory verbal hallucinations are related to cortical thinning in the left middle temporal gyrus of patients with schizophrenia. Psychol Med.

[40] Woodruff PW, Wright IC, Bullmore ET (1997). Auditory hallucinations and the temporal cortical response to speech in schizophrenia: a functional magnetic resonance imaging study. Am J Psychiatry.

[41] Holinger DP, Shenton ME, Wible CG, Donnino R, Kikinis R, Jolesz FA, McCarley RW (1999). Superior temporal gyrus volume abnormalities and thought disorder in left-handed schizophrenic men. Am J Psychiatry.

[42] Holt DJ, Kunkel L, Weiss AP (2006). Increased medial temporal lobe activation during the passive viewing of emotional and neutral facial expressions in schizophrenia. Schizophr Res.

[43] Goghari VM, Macdonald AW, Sponheim SR (2011). Temporal lobe structures and facial emotion recognition in schizophrenia patients and nonpsychotic relatives. Schizophr Bull.

[44] White T, Hongwanishkul D, Schmidt M (2011). Increased anterior cingulate and temporal lobe activity during visuospatial working memory in children and adolescents with schizophrenia. Schizophr Res.

[45] Oertel-Knöchel V, Knöchel C, Matura S, Prvulovic D, Linden DE, Van de Ven V (2013). Reduced functional connectivity and asymmetry of the planum temporale in patients with schizophrenia and first-degree relatives. Schizophr Res.

[46] Leroux E, Delcroix N, Dollfus S (2014). Left frontotemporal dysconnectivity within the language network in schizophrenia: an fMRI and DTI study. Psychiatry Res.

[47] Lavigne KM, Rapin LA, Metzak PD (2015). Left-dominant temporal-frontal hypercoupling in schizophrenia patients with hallucinations during speech perception. Schizophr Bull.

[48] Mondino M, Jardri R, Suaud-Chagny M, Saoud M, Poulet E, Brunelin J (2016). Effects of frontotemporal transcranial direct current stimulation on auditory verbal hallucinations and resting-state functional connectivity of the left temporoparietal junction in patients with schizophrenia. Schizophr Bull.

[49] Zhang L, Li B, Wang H (2017). Decreased middle temporal gyrus connectivity in the language network in schizophrenia patients with auditory verbal hallucinations. Neurosci Lett.

